# Cellular signaling and gene expression profiles evoked by a bivalent macrocyclic peptide that serves as an artificial MET receptor agonist

**DOI:** 10.1038/s41598-018-34835-4

**Published:** 2018-11-07

**Authors:** Wenyu Miao, Katsuya Sakai, Naoya Ozawa, Takumi Nishiuchi, Yoshinori Suzuki, Kenichiro Ito, Tomomi Morioka, Masataka Umitsu, Junichi Takagi, Hiroaki Suga, Kunio Matsumoto

**Affiliations:** 1 Division of Tumor Dynamics and Regulation, Cancer Research Institute, Kanazawa University, Kakuma, Kanazawa, 920-1192 Japan; 2 Department of Chemistry, Graduate School of Science, The University of Tokyo, Hongo, Bunkyo-ku, Tokyo, 113-0033 Japan; 3 Division of Functional Genomics, Advanced Science Research Center, Kanazawa University, Takaramachi, Kanazawa, 920-0934 Japan; 4 Laboratory of Protein Synthesis and Expression, Institute for Protein Research, Osaka University, Osaka, 565-0871 Japan; 5 Nano Life Science Institute, Kanazawa University, Kakuma, Kanazawa, 920-1192 Japan; 6 Institute for Frontier Science Initiative, Kanazawa University, Kakuma, Kanazawa, 920-1192 Japan

## Abstract

Non-native ligands for growth factor receptors that are generated by chemical synthesis are applicable to therapeutics. However, non-native ligands often regulate cellular signaling and biological responses in a different manner than native ligands. Generation of surrogate ligands comparable to native ligands is a challenging need. Here we investigated changes in signal transduction and gene expression evoked by a bivalent macrocyclic peptide (aMD5-PEG11) capable of high-affinity binding to the MET/hepatocyte growth factor (HGF) receptor. Binding of aMD5-PEG11 to the MET extracellular region was abolished by deletion of the IPT3−IPT4 domain, indicating the involvement of IPT3−IPT4 in the binding of aMD5-PEG11 to the MET receptor. aMD5-PEG11 induced dimerization and activation of the MET receptor and promoted cell migration that was comparable to induction of these activities by HGF. Signal activation profiles indicated that aMD5-PEG11 induced phosphorylation of intracellular signaling molecules, with a similar intensity and time dependency as HGF. In 3-D culture, aMD5-PEG11 as well as HGF induced epithelial tubulogenesis and up-regulated the same sets of functionally classified genes involved in multicellular organism development. Thus, a non-native surrogate ligand that consisted of a bivalent macrocyclic peptide can serve as an artificial MET receptor agonist that functionally substitutes for the native ligand, HGF.

## Introduction

Because growth factors and their receptors play critical roles in stem cell maintenance, tissue regeneration, and wound healing, growth factors have been developed as potent therapeutic drugs. Surrogate ligands capable of inducing receptor dimerization, e.g., monoclonal antibodies, engineered ligands, and other agents, have been generated^1–5^. For example, activation of the erythropoietin (EPO) receptor by synthetic peptides^6–8^ or by VH/VL variable domain fragments of anti-EPO receptor antibodies^5^ highlights the different dimer architectures of EPO receptors. Non-native ligands differentially regulate intracellular signaling and subsequent biological responses, often at different intensities and/or selectivities. Thus, receptor activation by antibodies or other surrogate ligands can produce fine-tuned and distinct receptor activation with biased downstream signaling. However, generation of surrogate ligands that achieve receptor activation and signal transmission that are comparable to that evoked by native ligands is challenging.

The MET receptor is a specific receptor for hepatocyte growth factor (HGF)^9,10^. Disruption of the gene encoding the MET receptor and therapeutic approaches with HGF have provided much evidence showing that MET receptor activation plays a definitive role in development, wound healing, regeneration, and anti-fibrosis^11,12^.

Recently, we generated macrocyclic peptides that function as artificial MET receptor agonists^13^. Macrocyclic peptides are appealing molecules for the discovery of molecular targeted drug candidates because of their unique characteristics, e.g., very high affinity to the target molecule, resistance to peptidases, feasibility of engineering, etc. We demonstrated that crosslinking of MET receptor-binding peptides identified by flexizyme-based cell-free translation and an mRNA display–based screening method (Random non-standard Peptide Integrated Discovery: RaPID) can generate agonistic ligands capable of inducing MET receptor activation and MET receptor-mediated biological responses^13^. However, the details of intracellular signaling and the gene expression profile of HGF compared to macrocyclic peptides that are MET receptor agonists have not been addressed.

If HGF and a MET receptor agonist that is a macrocyclic peptide evoke a similar intracellular signaling cascade, gene expression profile, and biological responses, RaPID-based macrocyclic peptide discovery and appropriate modification of bivalent/multivalent structures will provide a widely applicable strategy to obtain agonist macrocyclic peptides that substitute for growth factors and that activate cytokine receptors. In this study, we investigated changes in MET receptor dimerization, MET receptor activation, activation of intracellular signal transducers, and the gene expression profile that were triggered by HGF and the bivalent MET receptor agonist peptide.

## Results

### Mapping of the MET receptor binding domain

**Figure 1:**
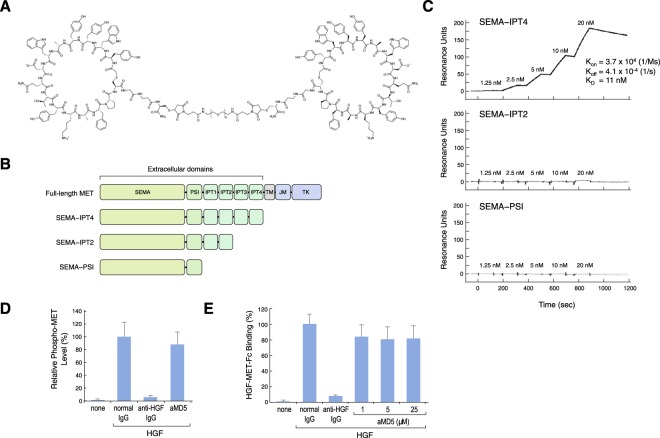
Mapping of the binding domain of aMD5-PEG11 in the MET receptor extracellular region. (**A**) Chemical structure of aMD5-PEG11. (**B**) Domain architecture of the MET receptor and its recombinant extracellular domains. (**C**) Association between aMD5 and MET receptor extracellular domains. Biotinylated aMD5 was immobilized on the sensor chip, and binding of increasing concentrations of various MET receptor-Fc proteins containing the indicated extracellular domains (SEMA–IPT4, SEMA–IPT2, and SEMA–PSI) was determined by SPR analysis. (**D**) Lack of an inhibitory effect of monomer aMD5 on HGF-induced MET receptor phosphorylation. Cells were cultured in the absence or presence of HGF (220 pM), anti-HGF IgG (100 nM), or aMD5 (1 μM). Each value indicates the mean ± SD obtained from independent experiments performed in triplicate. (**E**) Lack of a competitive inhibitory effect of monomer aMD5 on the binding of HGF to MET receptor-Fc. Binding of fluorescein-HGF (200 pM) to MET receptor-Fc immobilized on plates was measured in the absence or presence of anti-HGF IgG (100 nM) or aMD5 peptide. Each value indicates the mean ± SD obtained from independent experiments performed in triplicate.

The extracellular region of the MET receptor is composed of a semaphorin (SEMA) domain, a plexin-semaphorin-integrin (PSI) domain (similar in structure to the plexins, semaphorins and integrins), and four immunoglobulin-like plexin transcription (IPT) factor domains (similar in structure to the immunoglobulin-like fold shared by the plexins and transcription factors)^14,15^. The macrocyclic portion of aMD5-PEG11 is composed of 15 amino acids (Fig. 1A). aMD5 binds to the extracellular region of the MET receptor with a *K*_*D*_ value of 2.3 nM^13^; the binding region was not determined. To determine the binding domain of the MET receptor for aMD5, the association between aMD5 monomer and the full-length extracellular domain (SEMA–IPT4) and deleted MET receptor extracellular domains (SEMA–IPT2, SEMA–PSI) was analyzed with surface plasmon resonance (SPR) (Fig. 1B,C). A clear association between aMD5 and the full-length MET receptor extracellular region was seen at a *K*_*D*_ value of 11 nM, whereas the association was lost when the IPT3–IPT4 or IPT1–IPT4 domains were deleted from the full-length extracellular region (Fig. 1C). On the other hand, previous reports indicated that the SEMA domain is responsible for binding to HGF^16,17^. Therefore, the binding region in the MET receptor is different between HGF and aMD5-PEG11. Consistent with this, aMD5 neither competitively inhibited HGF-induced MET receptor phosphorylation in cultured cells (Fig. 1D) nor inhibited the protein-protein association between HGF and MET receptor-Fc (Fig. 1E).

### MET receptor dimerization and activation triggered by bivalent macrocyclic peptides

**Figure 2:**
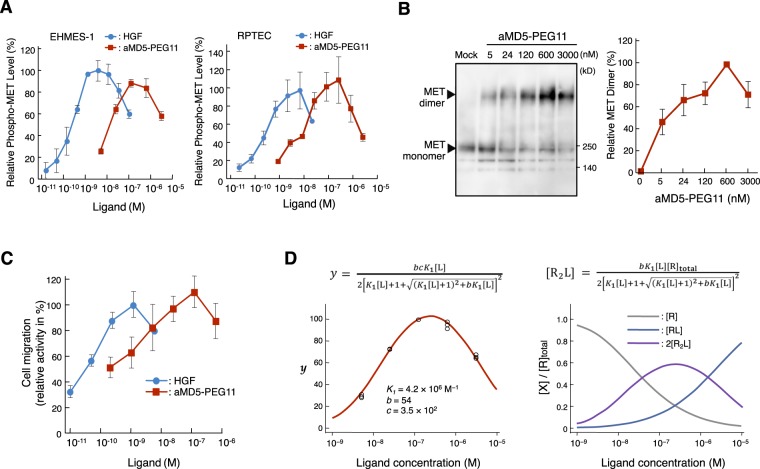
MET receptor dimerization, activation, and cell migration induced by aMD5-PEG11, and mathematical simulation analysis. (**A**) MET receptor activation. EHMES-1 cells and normal human renal proximal tubular epithelial cells (RPTEC) were treated with HGF or aMD5-PEG11, respectively, and the MET receptor Tyr 1234/1235 phosphorylation level was quantified with a cell-based enzyme-linked immunosorbent assay. Each value indicates the mean ± SD of triplicate measurements. (**B**) MET receptor dimerization induced by aMD5-PEG11. Cells were treated with cross-linker BS3, and MET receptor dimerization was analyzed by immunoprecipitation and western blotting using anti-MET antibody. The relative MET receptor dimer level was calculated by the band intensity in western blots. The SD was calculated from independent experiments performed in triplicate. (**C**) Cell migration promoted by aMD5-PEG11. Cells were stimulated with HGF or aMD5-PEG11 for 24 h, and cell migration was determined using the Oris assay. Each value indicates the mean ± SD obtained from independent experiments performed in triplicate. (**D**) Mathematical modeling of MET receptor activation. The MET activation level (red line) in the left graph was obtained by curve-fitting to the experimental values for aMD5-PEG11-induced MET receptor phosphorylation (open circles, n = 3) using the equation for *y*, and *K*_1_, *b*, and *c* were derived. Using these values and equations, changes in the relative abundance of [R] (monomeric MET receptor), [RL] (monomeric MET receptor bound with aMD5-PEG11), and [R_2_L] (dimeric MET receptor bound with aMD5-PEG11) were calculated.

Phosphorylation of Y1234/1235 residues within the MET receptor tyrosine kinase domain is an initial hallmark of MET receptor activation^18^. EHMES-1 and normal human renal proximal tubular epithelial cells (RPTEC) were stimulated with aMD5-PEG11 or HGF, respectively, and MET receptor activation was analyzed using a cell-based enzyme-linked immunosorbent assay, which selectively detects phosphorylated Y1234/1235 in the MET receptor (Fig. 2A). HGF triggered maximal MET receptor activation at concentrations of 1–10 nM, whereas MET receptor phosphorylation decreased from the maximal level with higher concentrations of HGF. aMD5-PEG11 increased MET receptor phosphorylation at higher concentrations compared with HGF. The maximal MET receptor activation by aMD5-PEG11 was seen at 120 nM, and the level was nearly comparable to that induced by HGF (Fig. 2A, left). MET receptor phosphorylation decreased from the maximal level with higher concentrations of aMD5-PEG11. Similar to the result in EHMES-1 cells, aMD5-PEG11 induced maximal MET receptor activation in a comparable manner as HGF and showed a bell-shaped profile in normal human renal proximal tubular epithelial cells (RPTEC) (Fig. 2A, right).

A critical first step for trans-phosphorylation of the MET receptor tyrosine kinase domain is considered to be dimerization of the MET receptor. We performed chemical cross-linking to analyze the MET receptor dimerization on the cell surface in cultured cells and detected MET receptor monomers and dimers with western blotting (Fig. 2B). Addition of aMD5-PEG11 induced dimerization of the MET receptor in a concentration-dependent manner. Maximal MET receptor dimerization was seen at 600 nM, whereas the level of dimerization clearly decreased at a higher concentration, showing a bell-shaped profile. Because MET receptor dimerization induced by aMD5-PEG11 showed similar concentration dependency and a bell-shaped profile as MET receptor tyrosine phosphorylation, the results indicate that MET receptor activation by aMD5-PEG11 is associated with the ability to induce dimerization of the MET receptor.

Cell migration induced by the HGF-MET receptor pathway plays a critical role in embryonic development and wound healing^19–23^. To determine if MET receptor activation by aMD5-PEG11 produced biological responses, we evaluated the ability of aMD5-PEG11 to induce cell migration. HGF potently facilitated migration of HuCCT1 cells in a dose-dependent manner (Fig. 2C). aMD5-PEG11 also facilitated cell migration with a maximal activity that was comparable to HGF. The comparable activity of aMD5-PEG11 to HGF seemed to be consistent with MET receptor activation.

Bell-shaped profiles for MET activation were also observed in previous studies^13,24,25^, suggesting a basic profile for receptor activation by bivalent ligands. [R_2_L], the concentration of dimerized receptor complexed with a bivalent ligand, changes according to the equation (Fig. 2D, right). Because activation of a receptor is proportional to [R_2_L], the active receptor level (*y*) is obtained as a linear function of [R_2_L] (Fig. 2D, left) (details to obtain equations are described in Materials and Methods). When a plot of experimentally obtained *y* values (MET tyrosine phosphorylation by cell-based enzyme-linked immunosorbent assay) against the ligand concentration [L] was curve-fitted with the equation, the parameters *K*_1_, *b*, and *c* could be obtained (Fig. 2D, left), and subsequently, changes in [R] (gray), [RL] (blue), and [R_2_L] (violet) could be calculated (Fig. 2D, right). An excess amount of ligand generates the inactive receptor-ligand complex [RL], and an increase in [RL] leads to a decrease in [R_2_L] at large excess ligand concentrations.

### Cellular signaling evoked by bivalent macrocycles

**Figure 3:**
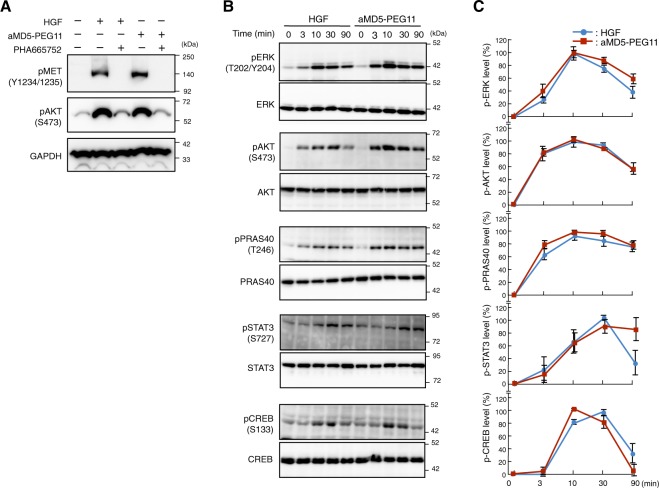
Time-dependent changes in the phosphorylation status of intracellular signaling molecules. (**A**) Selectivity of MET receptor activation by aMD5-PEG11. EHMES-1 cells were stimulated with 1 nM HGF or 120 nM aMD5-PEG11 in the absence or presence of the selective MET tyrosine kinase inhibitor, PHA665752 (100 nM) for 10 min. (**B**) Representative immunoblots for ERK, AKT, PRAS40, STAT3, and CREB. Total ERK, AKT, PRAS40, STAT3, and CREB levels were evaluated to ensure equal loading. (**C**) Changes in phosphorylation levels of ERK, AKT, PRAS40, STAT3, and CREB. EHMES-1 cells were stimulated with 1 nM HGF or 120 nM aMD5-PEG11 for 0, 3, 10, 30, or 90 min. Phosphorylation levels were expressed as the relative value in percent to the maximal level achieved by aMD5-PEG11. Each value indicates the mean ± SE obtained from independent experiments performed in triplicate.

MET receptor activation triggers downstream cellular signaling cascades involving phosphorylation of protein kinases. Previous studies demonstrated that HGF facilitates phosphorylation of a variety of kinases, including ERK1/2 and its downstream molecule CREB^26,27^, AKT and its downstream molecules such as PRAS40^28^, and STAT3^29^. PRAS40 Thr246 phosphorylation by AKT activates the mTORC1 pathway, thereby connecting the AKT and mTOR signaling pathways^30,31^. STAT3 and CREB are signal-transducing transcription factors involved in cell proliferation and survival^27,32,33^. Prior to detailed characterization and comparison of signal activation between aMD5-PEG11 and HGF, we confirmed the specificity of aMD5-PEG11 for the MET receptor by utilizing a selective inhibitor of the MET receptor. aMD5-PEG11 as well as HGF induced MET tyrosine phosphorylation and downstream AKT phosphorylation (Fig. 3A). The selective MET tyrosine kinase inhibitor PHA665752 almost completely blocked the activation of the MET receptor and AKT to basal levels. This result indicates that aMD5-PEG11 activates AKT through selective activation of the MET receptor.

To investigate the differences in characteristics of intracellular signal activation between aMD5-PEG11 and HGF, we assessed the time-dependent phosphorylation/activation involving ERK, AKT, PRAS40, STAT3, and CREB with western blotting (Fig. 3B,C). aMD5-PEG11 induced strong phosphorylation of ERK and AKT after 10 min, followed by a decrease over time. HGF induced similar time-dependent phosphorylation of ERK and AKT with a strength and profile comparable to aMD5-PEG11. Furthermore, the phosphorylation kinetics for the downstream molecules PRAS40, STAT3, and CREB induced by aMD5-PEG11 was also very similar to that obtained with HGF.

**Figure 4:**
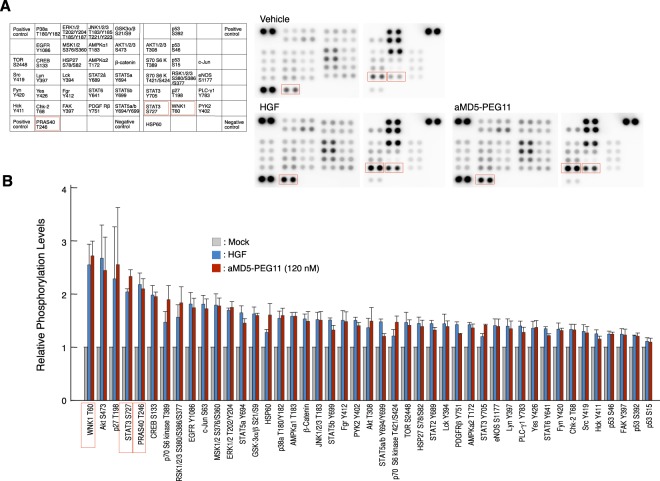
Changes in intracellular signaling activation by aMD5-PEG11. (**A**) Arrangement of proteins in the array (left) and representative pictures from array analysis (right). Positions of phosphoproteins showing remarkable increases upon aMD5-PEG11 or HGF stimulation are indicated by red squares. Cells were stimulated with 1 nM HGF or 120 nM aMD5-PEG11 for 10 min. (**B**) Changes in phosphorylation levels determined by quantitation of the protein phosphorylation array. Phosphoproteins showing remarkable increases upon aMD5-PEG11 or HGF stimulation are indicated by red squares. Each value indicates the mean ± SE obtained from independent experiments performed in triplicate. Representative immunoblots are shown.

To systematically assess the activation of cellular signaling through protein phosphorylation, we analyzed the changes in the phosphorylated signaling proteins in EHMES-1 cells treated with aMD5-PEG11 or HGF, using a phospho-kinase array (Fig. 4A). Both aMD5-PEG11 and HGF enhanced phosphorylation of a variety of signaling proteins, and clear enhancement was seen with STAT3, PRAS40, and WNK1 (Fig. 4A,B). Overall, we did not find signaling pathways that were selectively enhanced by either aMD5-PEG11 or HGF (Fig. 4B). Collectively, aMD5-PEG11 elicited activation/phosphorylation of a variety of intracellular signaling molecules at a similar strength and with similar kinetics as HGF.

### Changes in the gene expression profile

**Figure 5:**
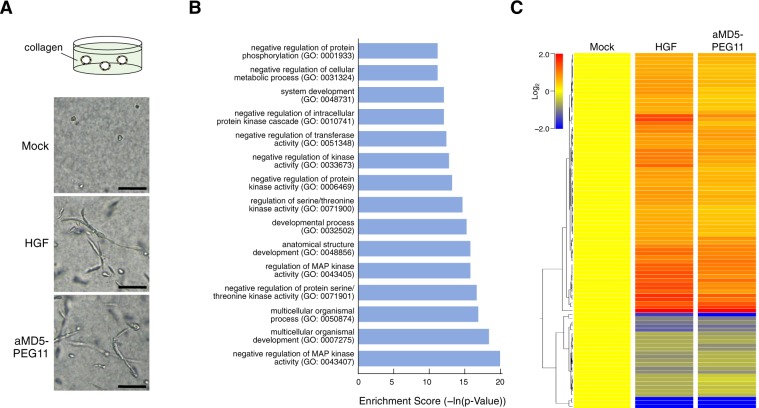
Changes in the gene expression profile induced by aMD5-PEG11. (**A**) Epithelial tubulogenesis induced by aMD5-PEG11 and HGF. Normal human renal proximal tubule epithelial cells were cultured in collagen gel in the absence or presence of aMD5-PEG11 or HGF. (**B**) The top 15 Gene Ontology terms significantly enhanced by aMD5-PEG11 and HGF. (**C**) Changes and comparison of gene expression profiles, as visualized by the heat map display, between control vehicle-treated cells, and cells treated with aMD5-PEG11 or HGF.

To further evaluate the functional compatibility between aMD5-PEG11 and HGF, the gene expression profile was analyzed with the Whole Human Genome Oligo Microarray. Because induction of epithelial tubulogenesis is unique to the HGF-MET receptor pathway^34^, we assayed this biological response to analyze the gene expression profile. When normal human renal proximal epithelial tubular cells were cultured in collagen gel for 8 days, aMD5-PEG11 as well as HGF induced tubulogenesis, indicating a MET receptor-mediated dynamic biological response (Fig. 5A).

To analyze changes in gene expression, the cells were cultured in collagen gel, treated with aMD5-PEG11 or HGF for 8 h, and total RNA was applied to the Microarray. Genes that showed a fold change larger than 1.5 (increase or decrease) were selected, and then further selected when the p value was less than 0.05 between two independent samples of HGF- and aMD5-PEG11-treated groups. Heat maps were prepared, and Gene Ontology analysis was performed based on these processed data. aMD5-PEG11 and HGF increased the same sets of functionally classified genes, including genes involved in multicellular organism development, anatomical structure development, multicellular organism processes, developmental processes, and system development (Fig. 5B) (raw data for gene ontology analysis are provided by Dataset 2). Because branching tubulogenesis is a key process for morphogenesis and organization of functional renal development, the result indicates that aMD5-PEG11 can orchestrate the gene expression network involved in a complex multicellular process, in a comparable manner to HGF. In addition to genes involved in development and morphogenesis, both aMD5-PEG11 and HGF increased expression of genes involved in negative regulation of protein kinases. Because MET receptor activation by aMD5-PEG11 and HGF triggered phosphorylation/activation of a variety of protein kinases (Fig. 4), the induction of these genes may represent a feedback mechanism following activation of multiple pathways. The heat map diagram indicates that aMD5-PEG11 increased or inhibited expression of a variety of genes (Fig. 5C) (raw data for the heatmap are provided by Dataset 1). Changes in several genes were distinguishable between aMD5-PEG11 and HGF. Overall, significantly up-regulated or down-regulated genes by aMD5-PEG11 overlapped those affected by HGF. We conclude that aMD5-PEG11 can elicit the authentic MET receptor signaling cascade and function as a mimetic of the physiological ligand, HGF.

## Discussion

Different types of non-native ligands capable of inducing dimerization/oligomerization of growth factor/cytokine receptors can evoke receptor-mediated signal activation. The apparent permissiveness and flexibility in dimer architecture needed for signal activation allowed us to test the possibility that different receptor-ligand dimer architectures can influence the signal strength and induce distinct signaling, leading to distinct biological responses. For example, covalently linked fragments of the VH/VL variable domain of antibodies against the extracellular domain of the EPO receptor generate agonistic bivalent molecules called “diabodies”. EPO receptors dimerized by diabodies with distinct epitopes elicit biased and distinct activation of signaling pathways compared to the native ligand, EPO^5^. The different dimer architectures and flexibility of growth factor/cytokine receptors may allow distinct signaling, as well as uneven signal strength.

A previous study showed that bivalent monoclonal antibodies with different epitopes in the extracellular domain of the MET receptor induce different biological responses: one monoclonal antibody enhances only cell motility, but the other monoclonal antibody triggers cell motility, proliferation, and epithelial tubulogenesis^35^. Covalent dimerization of NK1, the N-terminal and the first kringle domains of HGF, activates the MET receptor and enhances cell migration and proliferation with less potency compared to HGF^36^. NK2, another variant of HGF, promotes cell motility and survival but does not promote cell proliferation^37^. Artificial agonistic ligands for the MET receptor were generated by oligonucleotide-based screening^38^. The maximal activity of the oligonucleotide-based ligand exhibits maximal biological activities for enhanced MET receptor activation, cell proliferation, and migration, with a comparable ability as HGF. These results indicate that distinguishable activation of signaling molecules and subsequent biological responses can be elicited by distinct ligands that may induce different dimer/oligomer conformations of the MET receptor.

In the present study, we investigated the details of signal transduction and gene expression profiles evoked by the PEG-linked macrocyclic peptide, aMD5-PEG11. We obtained evidence that this PEG-linked bivalent macrocyclic peptide can induce activation of MET receptor-mediated signal transduction pathways and gene expression profile in a manner that was mostly indistinguishable from that of the native HGF protein. aMD5 binds to the structure determined by IPT3−IPT4 domains but does not bind to the SEMA domain to which HGF binds. The structural basis for MET receptor activation by the artificial surrogate ligand, aMD5-PEG11, and the native ligand, HGF, may be explained by several possibilities. MET receptor dimer conformation induced by aMD5-PEG11 may be either (i) similar to that induced by HGF even if the binding regions are different, or (ii) different from that induced by HGF, if the MET receptor has structural plasticity for dimer conformation that allows optimal/suboptimal tyrosine phosphorylation events in the MET receptor intracellular region, at least to some extent.

Tissue-specific disruption of the MET receptor revealed indispensable or important roles for the HGF-MET receptor pathway in the regeneration and protection of tissues, including the liver, kidney, skin, nervous system tissue, pancreas, etc^12^. Administration of HGF is a therapeutic option in preclinical disease models, including models of fulminant hepatitis, liver cirrhosis, amyotrophic lateral sclerosis, and spinal cord injury^11^. Cytokine and growth factor drugs exhibit distinct therapeutic actions because of their intrinsic biological activities, but they have general disadvantages in medical use. For instance, recombinant HGF rapidly disappears from circulating blood with a half-life of 3–5 min following intravenous administration^39^. Manufacturing of protein drugs is costly, whereas artificial growth factors/cytokines such as macrocycles can be manufactured by chemical synthesis. Because of their smaller size, they may show superior tissue permeability compared to protein drugs. Artificial MET receptor agonists such as macrocyclic peptides have the potential to be developed as novel biological drugs manufactured by chemical synthesis.

Finally, RaPID selection can be applied to various membrane proteins^40–43^, including other receptor tyrosine kinases, so that we will be able to obtain macrocycles that specifically bind such designated targets. As many of these transmembrane receptors are dimerized or heterodimerized following an interaction with their cognate ligand to activate signaling pathways^44,45^, our approach for generating dimeric macrocycles as non-protein ligands for cell surface receptors may be useful for developing surrogate ligands with a broad range of potential applications.

## Materials and Methods

### Cells, peptides, HGF, and reagents

The HuCCT1 human bile duct carcinoma cell line was obtained from the Japanese Cancer Research Resources Bank and cultured in RPMI-1640 medium supplemented with 10% fetal bovine serum (FBS) (Sigma-Aldrich). EHMES-1 human mesothelioma cells were cultured in RPMI-1640 medium supplemented with 10% FBS. Normal human renal proximal tubular epithelial cells were obtained from American Type Culture Collection (ATCC) and cultured in Renal Epithelial Cell Basal Medium (ATCC) supplemented with 0.5% FBS, 10 nM triiodothyronine, 10 ng/ml recombinant human epidermal growth factor, 100 ng/ml hydrocortisone hemisuccinate, 5 μg/ml recombinant human insulin, 1 μM epinephrine, 5 μg/ml transferrin, and 2.4 mM l-alanyl-l-glutamine. Recombinant human HGF (five amino acid deletion type) was prepared from the conditioned medium of CHO cells stably expressing human HGF. aMD5-PEG11 (Fig. 1A) and aMD5 monomer peptide were synthesized as described previously^13^. Anti-pERK (T202/Y204), -ERK, -pAKT (S473), -AKT, -pPRAS40 (T246), -PRAS40, -pSTAT3 (S727), -STAT3, -pCREB (S133), and -CREB antibodies were purchased from Cell Signaling.

### Truncated MET receptor extracellular proteins

For the expression of Fc-fused MET receptor extracellular domains, regions corresponding to residues 1–931 (SEMA–IPT4), 1–741 (SEMA–IPT2), and 1–563 (SEMA–PSI) were PCR amplified from the wild-type full-length human MET receptor cDNA and cloned into a pcDNA3.1-based vector containing the human IgG1 Fc fragment. The expression constructs were used to transiently transfect Expi293F cells (Thermo Fisher) using the ExpiFectamine 293 Transfection Kit (Thermo Fisher), and secreted proteins were purified from the culture supernatant using Protein A-Sepharose (GE Healthcare). All proteins were buffer exchanged to 20 mM Tris, 150 mM NaCl, pH 8.0, and concentrated to 0.2–0.5 mg/ml using an Amicon Ultra centrifugation device (Merck-Millipore, MWCO 30 kDa).

### SPR analysis

For biotin labeling of macrocycles, aMD5-Lys(Mmt)-NH-resin was synthesized by the Fmoc solid-phase peptide synthesis method, and the Mmt group was then deprotected by a solution of 98% dichloromethane, 1% trifluoroacetic acid, and 1% triisopropylsilane. The resulting aMD5-Lys-NH-resin was equilibrated with 20% *N,N*-diisopropylethylamine in *N*-methylpyrrolidone and treated with 0.2 M NHS-biotin in *N*-methylpyrrolidone. The modified peptide was deprotected/cleaved and macrocyclized, as described above, followed by purification with reverse-phase high-performance liquid chromatography and *in vacuo* lyophilization. C-terminal biotinylated macrocycles were captured on a streptavidin-immobilized SPR sensor chip at a surface density of 200–500 RU using a Biotin Capture Kit (GE Healthcare). The binding constants of the macrocycles to the MET receptor extracellular domains were determined by SPR analysis using Biacore T200 (GE Healthcare). The running buffer was HBS EP+ buffer (10 mM Hepes pH 7.4, 150 mM NaCl, 3 mM ethylenediaminetetraacetic acid, and 0.05% (v/v) SurfactantP20) containing 0.1% dimethylsulfoxide. MET receptor binding was tested by injecting varying concentrations of the MET receptor extracellular domain-Fc fusion proteins at 30 μl/min and quantifying with the single-cycle kinetics method. All data were fitted to the standard 1:1 binding model.

### Cell-based phospho-MET receptor measurement

EHMES-1 cells were seeded at 8 × 10^3^ cells per well in a 96-well black micro-clear plate (Greiner Bio-One) and cultured for 24 h. The cells were treated with HGF or MET receptor-binding dimeric macrocycle in RPMI-1640 medium supplemented with 10% FBS for 10 min, washed once with ice-cold phosphate-buffered saline (PBS), and fixed with 4% paraformaldehyde in PBS for 30 min at room temperature. After washing three times with PBS, the cells were blocked with 5% goat serum, 0.02% Triton X-100 in PBS for 30 min at room temperature and incubated with anti-phospho-MET (Tyr1234/1235) (D26) XP rabbit mAb (1:1000 in PBS with 1% goat serum) at 4 °C overnight. The cells were washed three times with PBS and incubated in horseradish peroxidase (HRP)-conjugated goat anti-rabbit antibody (1:1000 in PBS with 1% goat serum) for 1 h. After washing four times with PBS, tyrosine phosphorylated MET receptor was detected with ImmunoStar LD reagent (Wako) and measured using ARVO MX (Perkin Elmer). The relative MET receptor phosphorylation level was calculated as (sample chemiluminescence unit − mock control chemiluminescence unit)/(highest chemiluminescence unit − mock control chemiluminescence unit). Normal human renal proximal tubule epithelial cells were seeded at 2 × 10^4^ cells per well in a 96-well black micro-clear plate and cultured for 24 h. The cells were treated with aMD5-PEG11 or HGF for 10 min. The subsequent procedure was the same as described above.

### Binding assay between fluorescein-HGF and MET-receptor Fc beads

Fluorescein labeling of HGF was performed with a succinimidyl fluorescein labeling kit (Dojindo). Full-length MET receptor-Fc fusion protein (R&D Systems) was immobilized on high-binding 96-well plates (Corning) at 25 μg/ml, 100 μl/well in Tris-buffered saline (pH 7.5), 0.1% bovine serum albumin, and 0.05% Tween-20. The wells were washed three times, and fluorescein-HGF (200 pM) with or without rabbit polyclonal anti-HGF IgG^46^ (100 nM) or monomer aMD5 peptide (1, 5, 25 μM) was applied to wells containing immobilized MET receptor-Fc for 1 h at 25 °C. The wells were washed three times, and then fluorescent intensity of the wells was detected by using ARVO MX (Perkin Elmer).

### Analysis of MET receptor dimerization

EHMES-1 cells were seeded in 60-mm culture plates and cultured until about 90% confluent. Cells were washed twice with ice-cold RPMI-1640 supplemented with 10% FBS and incubated with dimeric macrocyclic peptides in RPMI-1640 containing 10% FBS for 60 min at 4 °C. Cells were washed with ice-cold PBS three times and treated with 1 mM bis (sulfosuccinimidyl) suberate (BS3, non-cell permeable cross-linker, Thermo Scientific) in PBS for 60 min at 4 °C. Non-reactive BS3 was quenched with 50 mM Tris (pH 8.0), 150 mM NaCl for 15 min at 4 °C. Cells were washed with ice-cold PBS and solubilized in lysis buffer (40 mM Tris-HCl (pH 8.0), 1% Triton X-100, 1% NP-40, 10% glycerol, 0.15 M NaCl, 2 mM EDTA, 1 mM phenylmethanesulfonyl fluoride, 1 × Complete protease inhibitor cocktail). Cell lysates were passed through a 27-G needle five times and centrifuged for 15 min at 15000 × *g*. Supernatants were incubated with 1 μg anti-MET antibody (Santa Cruz) for 4 h at 4 °C and incubated with 30 μl protein G-coupled magnetic beads (Dynabeads protein G, Invitrogen) for 12 h in a rotating apparatus. The beads were washed three times with lysis buffer and treated with SDS-PAGE Laemmli sample buffer supplemented with 5% mercaptoethanol. Samples were subjected to SDS-PAGE using a 5–20% gradient gel, and proteins were electroblotted to a PVDF membrane (Bio-Rad). The membrane was treated with anti-MET antibody (25H2, Cell Signaling), followed by HRP-conjugated anti-mouse IgG (Dako), and then visualized by chemiluminescent reaction using ARVO MX.

### Cell migration assay

Cell migration was measured using the Oris cell migration assay kit (Platypus Technology), according to the manufacturer’s protocol. Briefly, HuCCT1 cells were seeded at 2.5 × 10^4^ cells per well in a 96-well plate supplied in the kit and cultured for 24 h in RPMI-1640 supplemented with 10% FBS. The barrier was removed to reveal a void for migration of cells, and cells were further cultured in RPMI-1640 supplemented with 0.5% FBS in the absence or presence of dimeric macrocycle peptides or HGF for 24 h. Cells were washed with PBS and stained with 5 μg/ml calcein-AM at 37 °C for 15 min. Fluorescence intensity of the migrated cells was determined by ARVO MX.

### Mathematical modeling for receptor activation by a bivalent ligand

A model for complex formation between the receptor (R) and ligand (L) is expressed by two reactions:

1 $${\rm{R}}+{\rm{L}}\rightleftarrows {\rm{RL}}\,{K}_{1}$$

2 $${\rm{R}}+{\rm{RL}}\rightleftarrows {{\rm{R}}}_{2}{\rm{L}}\,{K}_{2}$$

where *K*_1_ and *K*_2_ are the equilibrium constants. Equilibrium equations are

3 $$\frac{[{\rm{RL}}]}{[{\rm{R}}][{\rm{L}}]}={K}_{1},\,\frac{[{{\rm{R}}}_{2}{\rm{L}}]}{[{\rm{R}}][{\rm{RL}}]}={K}_{2}$$

where [X] denotes the concentration of X. The mass balance equation for the receptor is

4 $$[{\rm{R}}]+[{\rm{RL}}]+2[{{\rm{R}}}_{2}{\rm{L}}]={[{\rm{R}}]}_{{\rm{total}}}$$

Here, the unit for [R], [RL], and [R_2_L] is mol dm^−2^, and the units for [L], *K*_1_, and *K*_2_ are mol dm^−3^, mol^−1^ dm^3^, and mol^−1^ dm^2^, respectively. Solving Equations 3 and 4 gives,

5 $$[{\rm{R}}]=\frac{2{[{\rm{R}}]}_{{\rm{total}}}}{{K}_{1}[{\rm{L}}]+1+\sqrt{{({K}_{1}[{\rm{L}}]+1)}^{2}+b{K}_{1}[{\rm{L}}]}}$$

6 $$[{\rm{RL}}]=\frac{2{K}_{1}[{\rm{L}}]{[{\rm{R}}]}_{{\rm{total}}}}{{K}_{1}[{\rm{L}}]+1+\sqrt{{({K}_{1}[{\rm{L}}]+1)}^{2}+b{K}_{1}[{\rm{L}}]}}$$

7 $$[{{\rm{R}}}_{2}{\rm{L}}]=\frac{b{K}_{1}[{\rm{L}}]{[{\rm{R}}]}_{{\rm{total}}}}{2{[{K}_{1}[{\rm{L}}]+1+\sqrt{{({K}_{1}[{\rm{L}}]+1)}^{2}+b{K}_{1}[{\rm{L}}]}]}^{2}}$$

where *b* = 8*K*_2_[R]_total_. If receptor activation is proportional to [R_2_L], the signal intensity *y*, which represents activation of the receptor, can be expressed using Equation 7 and a constant *a*, which indicates the efficiency of receptor activation upon R_2_L formation,

8 $$y=a[{{\rm{R}}}_{2}{\rm{L}}]=\frac{bc{K}_{1}[{\rm{L}}]}{2{[{K}_{1}[{\rm{L}}]+1+\sqrt{{({K}_{1}[{\rm{L}}]+1)}^{2}+b{K}_{1}[{\rm{L}}]}]}^{2}}$$

where *c* = *a*[R]_total_. Least squares fitting was performed using R software^47^.

### Western blot analysis

EHMES-1 cells were seeded at 9 × 10^5^ cells per well in 6-well plates and cultured overnight. To treat the cells with the MET receptor tyrosine kinase inhibitor, EHMES-1 cells were seeded at 3 × 10^5^ cells per well in 24-well plates and cultured with or without 100 nM PHA665752 for 12 h. After serum starvation for 4 h, cells were stimulated with HGF or dimeric macrocycle peptide. After washing with ice-cold PBS, cells were lysed in 200 μl 1 × SDS-PAGE Laemmli sample buffer and treated with ultrasonification (Vibra-cell). Cell lysates were analyzed by SDS-PAGE with a 7.5% polyacrylamide gel and electroblotted onto a PVDF membrane. The membrane was treated with primary antibodies (1:1000), followed by HRP-conjugated secondary antibodies (Dako) (1:2000). Chemiluminescence was visualized and quantitated using ImmunoStar LD (Wako). Original blots are provided by supplementary info.

### Phospho-kinase array

The phospho-kinase array was performed using the Proteome Profiler Human Phospho-Kinase Array Kit (R&D Systems). EHMES-1 cells were cultured at 1.5 × 10^6^ cells per plate in 60-mm dishes for 12 h. After serum starvation for 4 h, the cells were stimulated with dimeric macrocycle peptide or HGF for 10 min. Cells lysates were prepared, and phosphorylated protein kinases were detected, according to the manufacturer’s instructions. Chemiluminescence was detected using ImageQuant LAS 350 (GE Healthcare).

### Tubulogenesis assay

Normal human renal proximal tubule epithelial cells (2 × 10^4^ cells) were seeded in 400 μl Cellmatrix type IA collagen solution (Nitta Gelatin) in a 48-well plate and allowed to stand for 60 min at 37 °C for gelling, according to the manufacturer’s instructions. After gelation, 500 μl renal epithelial basal medium supplemented with 0.5% FBS and 2.4 mM l-alanyl-l-glutamine, with or without HGF or dimeric macrocycle peptide, was added to each well. Cells were cultured for 8 days, and the culture medium (with or without HGF or aMD5-PEG11) was refreshed every 2 days.

### mRNA profiling array

For the mRNA array, normal human renal proximal tubule epithelial cells were seeded at 2 × 10^5^ cells per well in 1.2 ml Cellmatrix type IA collagen solution (Nitta Gelatin) in a 12-well plate and allowed to stand for 60 min at 37 °C for gelling, according to the manufacturer’s instructions. Culture medium (800 μl) supplemented with 0.5% FBS and 2.4 mM l-alanyl-l-glutamine was added, and the cells were cultured for 12 h. Then, the cells were cultured in the absence or presence of dimeric macrocycle peptide or HGF for 8 h. Cells were harvested, and total RNA was prepared using Seposal-RNA I Super G (Nacalai Tesque), according to the manufacturer’s instructions. Microarray analyses were performed using the Whole Human Genome (4 × 44 k, G2565BA) Oligo Microarray, according to the Agilent 60-mer Oligo Microarray Processing Protocol (Agilent Technologies). Total RNA samples (200 ng) were used to prepare Cy3-labeled cRNA using a Low RNA Input Fluorescent Linear Amplification Kit (Agilent Technologies). Fluorescence-labeled cRNAs were purified using an RNeasy RNA Purification Kit (Qiagen Inc., Hilden, Germany). RNA samples obtained by duplicate experiments performed independently were used to confirm the reproducibility of the microarray analyses. The images were analyzed using Feature Extraction Software (Ver. 10.7.3.1) and GeneSpring GX 11.5 software (Agilent Technologies). Normalization was performed as follows: (i) intensity-dependent Lowess normalization; (ii) data transformation, with measurements set to ≤0.01; (iii) per-chip 75^th^ percentile normalization of each array; and, (iv) per-gene: normalized to the median of each gene. Genes with more than a 1.5-fold difference in expression between aMD5-PEG11-treated and HGF-treated groups were selected and used for the Gene Ontology enrichment analysis, which was performed using DAVID Bioinformatics Resources 6.7. The raw and processed data were deposited in the Gene Expression Omnibus database (access ID: GSE111542).

## Electronic supplementary material

Supplementary information

Dataset 1

Dataset 2
