# Supplementary material for: Cellular signaling and gene expression profiles evoked by a bivalent macrocyclic peptide that serves as an artificial MET receptor agonist
Source: Sci Rep. 2018 Nov 7;8:16492. doi: 10.1038/s41598-018-34835-4 (PMC6220203; doi:10.1038/s41598-018-34835-4)
Supplement: Supplementary file 1 — Supplementary information [file 41598_2018_34835_MOESM1_ESM.pdf]

Cellular signaling and gene expression profiles evoked by a bivalent macrocyclic peptide that serves as an artificial MET receptor agonist

Wenyu Miao, Katsuya Sakai, Naoya Ozawa, Takumi Nishiuchi, Yoshinori Suzuki, Kenichiro Ito, Tomomi Morioka, Masataka Umitsu, Junichi Takagi, Hiroaki Suga and Kunio Matsumoto

Supplementary Figure 1

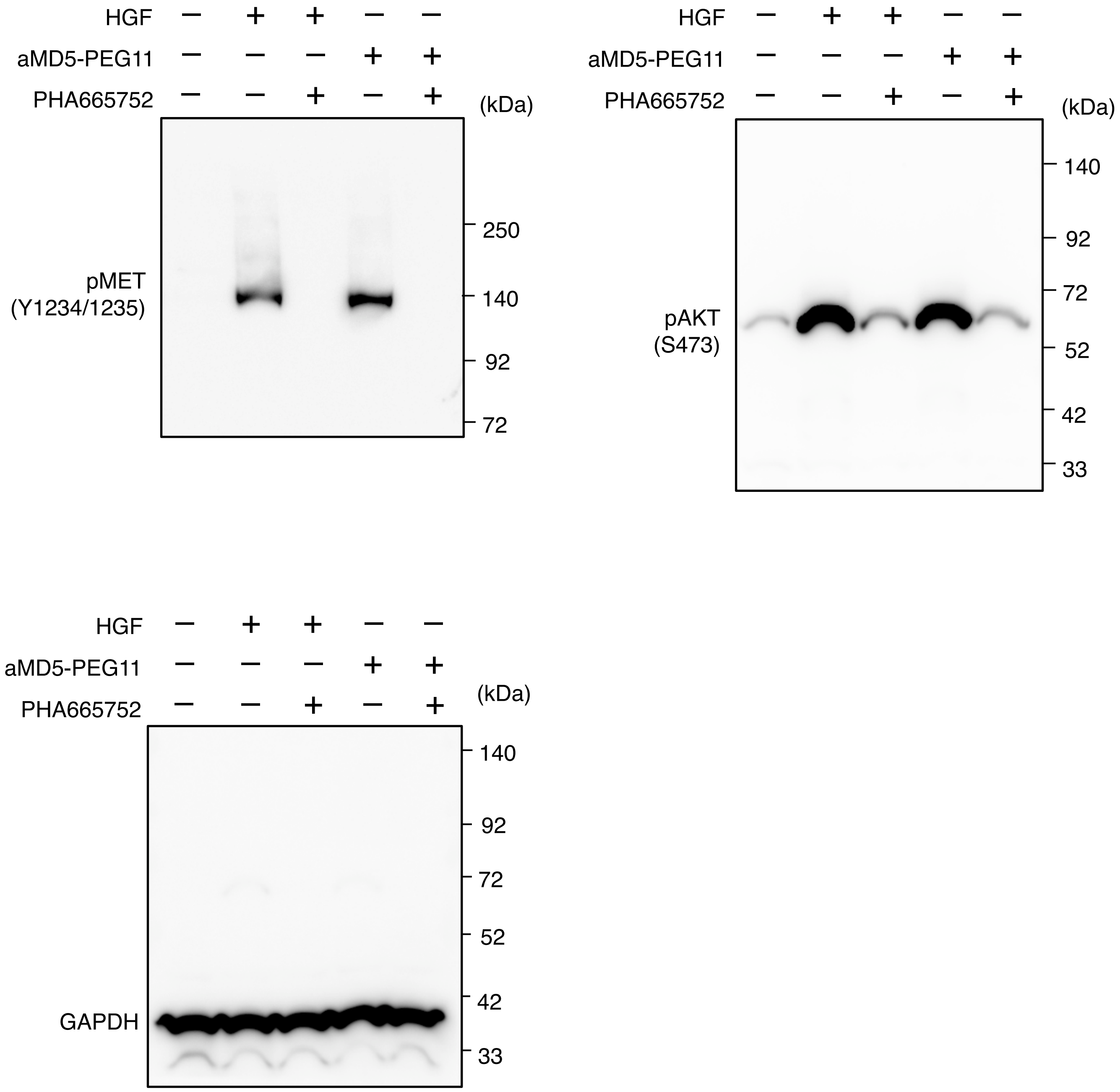

Supplementary Fig.1. Original blot images for Fig. 3A.

Supplementary Figure 2

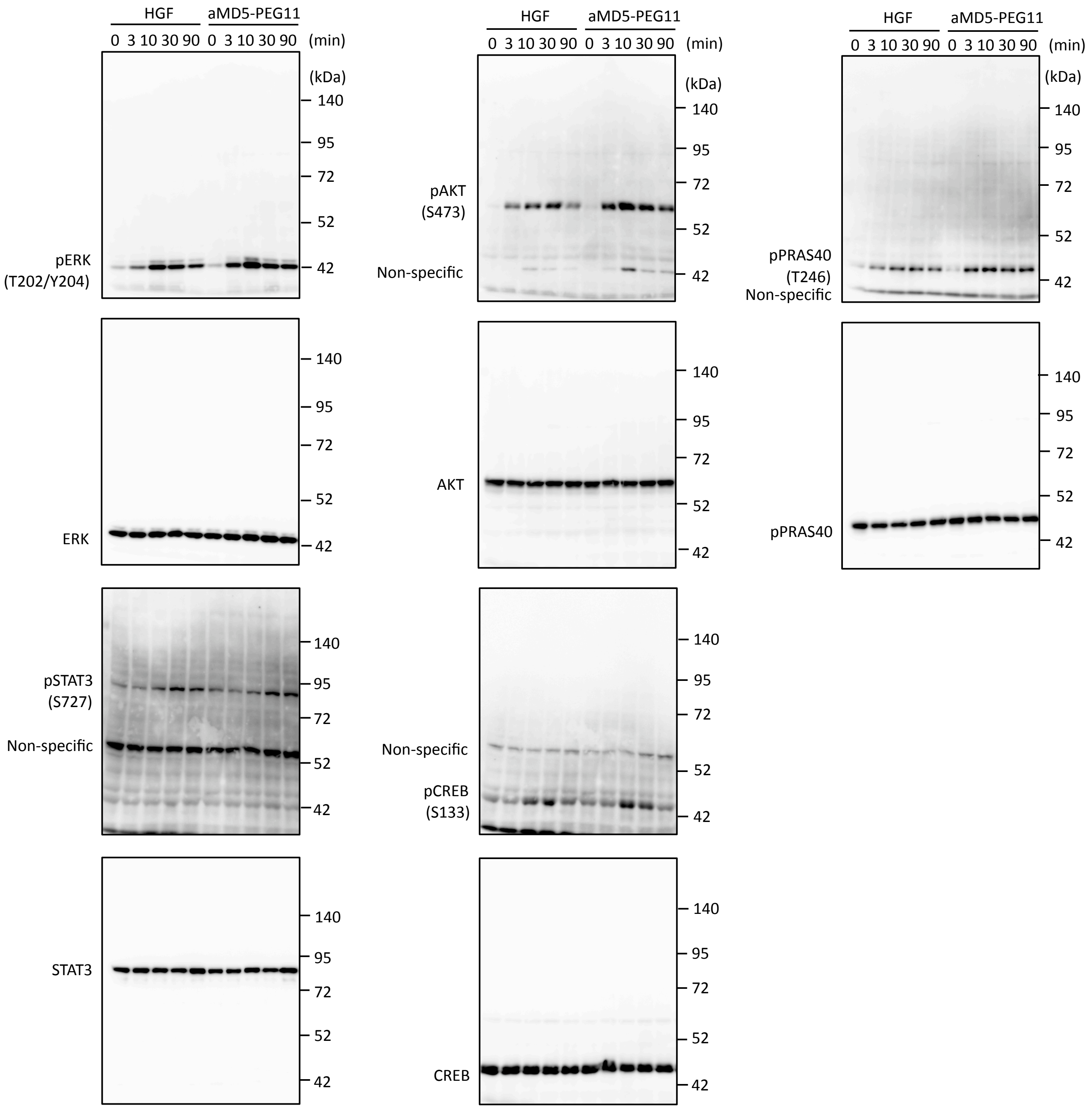

Supplementary Fig.2. Original blot images for Fig. 3B.
